# A Descriptive Analysis of Men Diagnosed With Epididymitis, Orchitis, or Both in the Emergency Department

**DOI:** 10.7759/cureus.15800

**Published:** 2021-06-21

**Authors:** Mason Bonner, Johnathan M Sheele, Santiago Cantillo-Campos, Justin M Elkins

**Affiliations:** 1 Emergency Medicine, Mayo Clinic, Jacksonville, USA

**Keywords:** epididymitis, orchitis, epididymo-orchitis, chlamydia

## Abstract

Introduction

Epididymitis and orchitis are illnesses characterized by pain and inflammation of the epididymis and testicle. They represent the most common causes of acute scrotal pain in the outpatient setting. Epididymitis and orchitis have both infectious and noninfectious causes, with most cases being secondary to the invasive pathogens chlamydia, gonorrhea, and *Escherichia coli* (*E.coli)*. The study's objective was to examine the epidemiology and clinical characteristics of men diagnosed with epididymitis or orchitis in a United States emergency department.

Methods

We examined a dataset of 75,000 emergency department (ED) patient encounters from a single health system in Northeast Ohio who underwent nucleic acid amplification testing (NAAT) for chlamydia, gonorrhea, or trichomonas, or who received a urinalysis and urine culture. All patients were ≥18 years of age, and all encounters took place between April 18, 2014, and March 7, 2017. The analysis only included men receiving an ED diagnosis of epididymitis, orchitis, or both. We evaluated laboratory and demographic data using univariable and multivariable analyses.

Results

There were 1.3% (256/19,308) of men in the dataset diagnosed with epididymitis, orchitis, or both. Only 50.1% (130/256) of men diagnosed with epididymitis, orchitis, or both were tested for gonorrhea and chlamydia during their clinical encounter, and among those 13.8% (18/130) were positive. Chlamydia (12.3% [16/130]) was more common than both gonorrhea (3.1% [4/129]) and trichomonas (8.8% [3/34]) among men <35 years of age diagnosed with epididymitis, orchitis, or both. Only 62.1% of men diagnosed with epididymitis, orchitis, or both received a urine culture, of which 20.1% grew bacteria at ≥10,000 CFU/ml. *E. coli* (N= 20) was the most common bacteria growing in urine culture followed by *Streptococcus *(N= 3), *Klebsiella *(N= 2), *Pseudomonas *(N= 2), and *Serratia *(N= 2). Men diagnosed with epididymitis, orchitis, or both who had a positive urine culture were more likely to be ≥35 years of age, married, had higher urine white blood cells (WBCs), more urine bacteria, higher urine leukocyte esterase, more likely to have urine nitrite, and were less likely to be empirically treated for gonorrhea and chlamydia (*P*≤.03 for all).* *

Conclusions

In the ED, epididymitis, orchitis, or both are uncommonly diagnosed among patients undergoing genitourinary tract laboratory testing. Sexually transmitted infections (STIs) are common in men <35 years of age diagnosed with epididymitis, orchitis, or both, with chlamydia being most common. *E. coli* was the most common bacteria growing in urine culture.

## Introduction

Epididymitis and orchitis are characterized by pain and inflammation of the epididymis and testicle, respectively. They can occur together as epididymo-orchitis or independently. There are roughly 600,000 cases of epididymitis annually in the United States. It affects all age groups and represents the most common cause of acute scrotal pain in the outpatient setting [1,2]. The etiology, diagnostic evaluation, and treatment differ depending upon the patient's age [3,4]. 

Acute epididymitis is a disease isolated to the epididymis; however, it occurs in concert with orchitis in 47-58% of cases [5,6]. Isolated orchitis without epididymis is quite uncommon and is typically caused by non-sexually transmitted infections (STIs) such as the Coxsackie-B virus, mumps, or through the hematogenous spread of bacteria [7]. Epididymo-orchitis typically begins as unilateral scrotal tenderness that worsens over several days and can advance to include generalized and bilateral testicular tenderness, testicular swelling, reactive hydrocele, dysuria/urethral syndromes, and overlying erythema [8,9]. Severe cases may present with fever, nausea, and systemic symptoms. The pathophysiology of infectious epididymitis is still not completely understood but may result from reflux of infected urine into the ejaculatory duct secondary to an obstructed outlet [3,10]. Processes such as benign prostatic hypertrophy may play a role via outlet obstruction in developing this disease [3,10].

Epididymitis, epididymo-orchitis, and orchitis are known to have both infectious and noninfectious causes, and routine screening has only identified a bacterial cause in 25% of cases [10]. For men < 35 years of age, the most common causes of epididymitis and epididymo-orchitis are chlamydia and gonorrhea [11,12]. Men over 35 years of age with epididymitis are more likely to be infected with coliform bacteria, with *E. coli* being the most common [13-15]. Other rare infectious causes of epididymitis include tuberculosis and brucellosis [16-17]. Noninfectious causes of the disease include genitourinary trauma, iatrogenic bladder or urethral instrumentation, amiodarone, and autoimmune illnesses such as sarcoidosis and Behçet syndrome [18-20]. 

There is a paucity of large-scale studies evaluating patients' clinical characteristics with epididymitis, orchitis, or both in the emergency department (ED). This study aims to examine the epidemiology and clinical characteristics of men with epididymitis, orchitis, or both in the ED, specifically those with and without a sexually transmitted infection (STI) or a urinary tract infection (UTI).

## Materials and methods

The study received institutional review board approval from University Hospitals (UH). The UH information technology (IT) team created a database consisting of 75,000 UH emergency department (ED) patient encounters in Northeast Ohio between April 18, 2014, to March 7, 2017. Data were extracted from the UH electronic medical record using a custom structured query language (SQL) in SQL Server Management Studio (SSMS). All patients were ≥18 years of age, seen in a UH ED, and underwent testing for gonorrhea, chlamydia, trichomonas, or had a urinalysis and urine culture performed. We used the existing dataset to explore our clinical question and only included men in the analysis. Analyses from the dataset have previously been published [21-25].

Men with epididymitis, orchitis, or both had been combined into a single variable by UH IT when the dataset was created using the following ED International Classification of Diseases, Ninth Revision (ICD-9), and International Statistical Classification of Diseases, Tenth Revision (ICD-10) discharge codes: N45, N45.1, N45.4, 604.0, 604.90, or 604.99. Patients were diagnosed with a urinary tract infection (UTI) if they had one of the following ED discharge (ICD-9, -10) codes: N30.0, N30.00, N30.01, N30, N30.9, N30.90, N39.0, O03.38, O03.88, O04.88, O08.83, O23.10, O23.40, O86.2, O86.20, O86.22, O86.29, 595.0, 595.89, 595.9, 599.0, 639.8, 646.60, or 646.64. Men were diagnosed with prostatitis if they had an (ICD-9, -10) code of N41, N41.0, N41.8, N41.9, N42, 601.0, 601.8, or 601.9. Patients were infected with N gonorrhoeae and C trachomatis if they had a positive nucleic acid amplification test (NAAT) (APTIMA, Hologic). Patients were infected with Trichomonas vaginalis if the organism was seen on genital wet prep, reported on the urinalysis, or had a positive NAAT (APTIMA, Hologic). Patients had to have a negative T vaginalis NAAT to be classified as uninfected. Trace urine protein was categorized as 0.5 mg/dL for the analysis. To account for differences in how urine red blood cells (RBCs) and white blood cells (WBCs) were reported from the laboratory, all urine RBCs and WBCs >100 cells/HPF were recorded as 101, and for any ranges of urine RBCs and WBCs, the mean of that range was used for the analysis. A urine culture growing ≥10,000 colony forming units (CFU)/mL was considered positive, and <10,000 CFU/mL was negative. Men given ceftriaxone or cefixime plus azithromycin or an outpatient prescription for doxycycline were considered treated for gonorrhea and chlamydia. Missing and erroneous variables were not included in the analysis.

Data analysis

Continuous variables were summarized as the median and interquartile range (IQR) and were analyzed using the Wilcoxon rank-sum test. Categorical variables were summarized as counts and percentages and analyzed using the Chi-square test. Unless otherwise stated all multivariable logistic regression accounted for: age (years), black race vs. other race, urine leukocyte esterase (0-3+), urine WBCs (0-101), tested for gonorrhea and chlamydia (vs not), urine protein (0, 0.5 (trace), 1+, 2+, or 3+), urine bacteria (0-4+), urine RBCs (0-101), urine urobilinogen (0, 2, 4, 8, or 12), urine blood 0-3+), and marital status (married, single, or divorced, widowed, or separated). Odds ratios and 95% confidence intervals were calculated, and a P-value of <.05 was considered statistically significant. The analysis was conducted using JMP Pro 14 (SAS Institute Inc).

## Results

Epididymitis, orchitis, or both were diagnosed in 256/19,308 (1.3%) ED encounters. The clinical characteristic for men in the dataset with and without epididymitis, orchitis, or both are summarized in Table 1. On univariable analysis, men with epididymitis, orchitis, or both, compared to men in the dataset without these diagnoses, were younger, less likely to arrive by emergency medical services (EMS), less likely to be admitted to the hospital, were more likely to be single, less likely to have a primary care physician, and had higher triage pain scores (P≤.002). On regression analysis, men with epididymitis, orchitis, or both were significantly younger, less likely to arrive by EMS, less likely to be admitted to the hospital, less likely to have a primary care physician, have higher ED triage pain scores, and have lower emergency severity index (ESI) scores (P≤.006 for all). On univariable analysis, there were significant differences in the urinalysis between those with and without epididymitis, orchitis, or both, including the presence of bacteria, blood, protein, RBCs, urobilinogen, WBCs (P≤.02). On regression analysis, only urine WBCs were significantly higher for those with epididymitis, orchitis, or both (P=.03). Men with epididymitis, orchitis, or both were more likely tested for gonorrhea and chlamydia and less likely to be diagnosed with a UTI (P≤.001).

**Table 1:** Demographic and clinical characteristics of men with and without epididymitis or orchitis.

	+Epididymitis and/or orchitis (N=256)	No epididymitis or orchitis (N=19,052	p-value	Adjusted OR (95% CI) for having epididymitis and/or orchitis	Adjusted p-value
Age	35 (25,53)	61 (38,77)	<.001	.14 (.97-.99)	<.001
Black race, %	57% (145/253)	48% (8,999/18,957)	.002	.88 (.59-1.31)	.52
Arrived by EMS (vs. not)	5% (12/254)	38% (7,229/18,812)	<.001	.12 (.06-.26)	<.001
Admitted from the ED	17% (43/256)	47% (8,859/19,052)	<.001	.50 (.32-.77)	.002
Marital status Single Married Divorced, widowed, separated	67% (171/255) 25% (63/255) 8% (21/255)	44% (8,295/18,934) 40% (7,489/18,934) 17% (3,150/18,934)	<.001	NA	NA
+ Primary care physician	25% (63/256)	42% (8,095/19,052)	<.001	.55 (.36-.84)	.006
Triage pain scale	5 (0,7) N= 48	0 (0,5) N= 8,468	<.001	1.17 (1.06-1.28)	.002
Hour of ED visit	14 (10,18)	14 (10,18)	.44	1.00 (.98-1.03)	.78
ED encounter over weekend (vs weekday)	25% (64/256)	28% (5,277/19,052)	.34	.87 (.60-1.27)	.48
Emergency severity index (ESI)	3 (3,3) N= 251	3 (3,3) N=18,387	.14	.57 (.43-.74)	<.001
Urine source: Clean catch Straight catheter Old bladder catheter Unknown	94% (124/132) 2% (3/132) 4% (5/132)	66% (7,924/11,983) 6% (749/11,983) 28% (3,310/11,983)	<.001	NA	NA
Urine bacteria	0 (0,1) N= 149	1 (0,2) N= 12,178	.002	.91 (.77-1.08)	.28
Urine blood	0 (0,1) N= 231	0 (0,2) N= 15,814	<.001	.97 (.78-1.21)	.79
Urine leukocyte esterase	0 (0,2) N= 233	0 (0,2) N= 15,902	.28	1.16 (.94-1.44)	.17
Urine nitrite positive (vs negative)	7% (16/234)	8% (1,241/16,122)	.62	1.46 (.20-.82)	.20
Urine protein	0 (0,1) N= 234	1 (0,2) N= 16,060	<.001	.81 (.65-1.01)	.06
Urine RBCs	3 (3,13) N= 151	3 (3,36) N= 12,166	.02	1.00 (.99-1.01)	.55
Urine Urobilinogen	0 (0,2) N= 234	0 (0,0) N= 16,128	.02	1.04 (.95-1.14)	.44
Urine WBCs	18 (2.5,101) N= 151	13 (3,45) N= 12,164	<.001	1.01 (1.00-1.01)	.03
Urine culture ≥10,000 CFU/mL, %	20% (32/159)	22% (3,176/14,589)	.70	1.25 (.73-2.12)	.42
Tested for gonorrhea and chlamydia	51% (130/256)	16% (3,002/19,052)	<.001	3.94 (2.47-6.29)	<.001
Diagnosed with a UTI	5% (12/256)	13% (2,527/19,052)	<.001	.27 (.14-.50)	<.001
Diagnosed with prostatitis	0% (0/256)	.4% (77/19,052)	.31	NA	NA

Epididymitis, orchitis, or both compared to men with UTI

There were 2,539/19,308 (13.1%) men in the dataset diagnosed with a UTI. Among those men diagnosed with a UTI and no epididymitis or orchitis, 93% (2,362/2,527) had a urine culture performed, and 51% (1,194/2,362) grew bacteria at ≥10,000 CFU/mL. Men with epididymitis, orchitis, or both, when compared to men diagnosed with a UTI but not epididymitis or orchitis, were younger, more likely Black, less likely married, had fewer urine WBCs, fewer urine RBCs, fewer urine bacteria, less urine protein, less urine leukocyte esterase, and less urine nitrite (P<.001 for all) (Table 2). On multivariable analysis, those with epididymitis, orchitis, or both were significantly younger, had fewer urine bacteria, and had lower urine leukocyte esterase than men diagnosed with a UTI (P<.001). There were 146/2,527 (5.8%) of men diagnosed with a UTI but not with epididymitis or orchitis tested for gonorrhea and chlamydia. Among these, 41.8% (61/146) were positive for gonorrhea, chlamydia, or both. In comparison, 51.6% (126/244) of men with epididymitis, orchitis, or both were not diagnosed with a UTI and underwent testing for gonorrhea and chlamydia. Among these, 12.7% (16/126) were positive for either or both infections.

**Table 2:** Comparison of men with epididymitis and/or orchitis and men diagnosed with a UTI. NG: Neisseria gonorrhoeae, CT: Chlamydia trachomatis,

	+Epididymitis and/or orchitis (N=256)	+UTI and no epididymitis or orchitis (N=2,527)	p-value	Adjusted OR (95% CI) for those with epididymitis and/or orchitis	Adjusted p-value
Age	35 (25, 53)	71 (54,83)	< .001>	.98 (.97-.99)	< .001>
Black race, %	57% (145/253)	40% (1015/2522)	< .001>	1.13 (.74-1.72)	.57
Marital status (% married)	25% (63/255)	45% (1126/2513)	< .001>	NA	NA
Urine WBCs	18 (3,101) N= 151	60 (13,101) N= 2442	< .001>	1.00 (1.00-1.01)	.33
Urine RBCs	3 (3,13) N= 151	13 (3,37) N= 2449	< .001>	.99 (.99-1.00)	.21
Urine bacteria	0 (0,1) N= 149	1 (1,3) N= 2479	< .001>	.74 (.62-.88)	< .001>
Urine protein	0 (0,1) N= 234	1 (1,3) N= 2475	< .001>	.89 (.71-1.12)	.34
Urine leukocyte esterase	0 (0,2) N= 233	3 (1,3) N= 2391	< .001>	.69 (.55-.85)	< .001>
Urine nitrite	7% (16/234)	22% (536/2492)	< .001>	.93 (.51-1.68)	.80
Urine culture growing ≥10,000 CFU/mL bacteriuria	20% (32/159)	51% (1194/2362)	< .001>	1.10 (.66-1.81)	.72
+Gonorrhea	3% (4/129)	24% (35/145)	< .001>	.26 (.08-.86)	.03
+Chlamydia	12% (16/130)	24% (35/146)	.01	.88 (.40-1.92)*	.75*
+Trichomonas	9% (3/34)	6% (2/31)	.72	.96 (.04-21.82)*	.98*
+Any STI	15% (20/130)	43% (63/146)	< .001>	.48 (.22-1.02)*	.05*

Epididymitis, orchitis, or both in men with and without concurrent UTI

There were 4.7% (N=12) men diagnosed with epididymitis, orchitis, or both and a concurrent UTI; however, only eight received a urine culture, and of those four grew bacteria at ≥10,000 CFU/mL. Among the 12 men with epididymitis, orchitis, or both and diagnosed with a UTI, one had chlamydia, one had gonorrhea, and none were positive for trichomonas. Men with epididymitis, orchitis, or both and a UTI diagnosis had higher urine WBCs, higher median urine blood, higher median leukocyte esterase, proteinuria, and were more likely to have a positive urine culture (P≤.03) (Table 3).

**Table 3:** Comparison of men with epididymitis and/or orchitis and diagnosed with a UTI compared to those diagnosed with epididymitis and/or orchitis and without concurrent UTI.

	+Epididymitis and/or orchitis and +UTI (N=12)	+Epididymitis and/or orchitis and -UTI (N= 244)	p-value
Age	51 (29,62)	35 (25,52)	.22
Black race, %	50% (6/12)	58% (139/241)	.60
Marital status (% married)	50% (6/12)	23% (57/243)	.09
Urine WBCs	76 (36,101)	13 (3,101) N= 139	.01
Urine RBCs	9 (3,43)	3 (2,13) N= 139	.08
Urine blood	1 (1,2) N= 11	0 (0,1) N= 220	.002
Urine bacteria	1 (0,1)	0 (0,1) N= 137	.14
Urine leukocyte esterase	3 (2,3) N= 11	0 (0,2) N= 222	<.001
Urine nitrite	0% (0/12)	7% (16/222)	.34
Urine protein	2 (0,2)	0 (0,1) N= 222	.004
Urine culture growing ≥10,000 CFU/mL bacteriuria	50% (4/8)	19% (28/151)	.03
Tested for gonorrhea and/or chlamydia	33% (4/12)	52% (126/244)	.22
+Gonorrhea	25% (1/4)	2% (3/125)	.01
+Chlamydia	25% (1/4)	12% (15/126)	.43
+Trichomonas	0% (0/1)	9% (3/33)	.75
+ Any STI	50% (2/4)	14% (18/126)	.05

Epididymitis, orchitis, or both and urine cultures

There were 62.1% (N=159) men diagnosed with epididymitis, orchitis, or both that had a urine culture performed, of which 20.1% (32/159) grew bacteria at ≥10,000 CFU/mL (Table 4). The most common bacterial genus identified in men with epididymitis, orchitis, or both were: Escherichia (N=20), Streptococcus (N=3), Klebsiella (N=2), Pseudomonas (N=2), Serratia (N=2), Staphylococcus (N=1), Lactobacillus (N=1), Enterococcus (N=1). No patient with epididymitis, orchitis, or both and a positive urine culture also had a positive test for gonorrhea, chlamydia, or trichomonas. On univariable analysis, men with epididymitis, orchitis, or both and positive urine culture were significantly older, more likely married, had higher urine WBCs, more urine RBCs, more urine blood, more urine bacteria on urinalysis, higher protein, and were more likely to be nitrite positive (P≤.04 for all) (Table 4). On multivariable regression analysis, men with epididymitis, orchitis, or both and positive urine culture were significantly older, more likely to be Black, had higher urine bacteria, and more likely to have positive nitrite urine (P≤.02 for all).

**Table 4:** Men with epididymitis and/or orchitis and a positive urine culture compared to men with epididymitis and/or orchitis and a negative urine culture.

	+Epididymitis and/or orchitis and +urine culture (N=32)	+Epididymitis and/or orchitis and -urine culture (N=127)	p-value	Adjusted OR (95% CI) for those with a positive urine culture	Adjusted p-value
Age	65 (47,77)	37 (25, 53)	<.001	1.08 (1.03-1.15)	.004
Black race, %	63% (20/32)	47% (59/125)	.12	10.34 (1.42-74.97)	.02
Marital status (% married)	50% (16/32)	27% (34/126)	.04	NA	NA
Urine WBCs	101 (41,101) N= 31	13 (3,76) N= 78	<.001	1.03 (.99-1.06)	.08
Urine RBCs	11 (3,24) N= 31	3 (3,13) N= 78	.01	1.00 (.97-1.02)	.86
Urine blood	1 (1,2)	0 (0,1) N= 120	<.001	.62 (.25-1.36)	.26
Urine bacteria	1 (1,3) N= 31	0 (0,1) N=77	<.001	2.31 (1.38-4.22)	.003
Urine protein	1 (0,2)	0 (0,1) N= 122	.003	.59 (.21-1.49)	.28
Urine leukocyte esterase	3 (2,3)	0 (0,2) N= 121	<.001	1.50 (.65-3.67)	.35
Urine nitrite	34% (11/32)	1% (1/122)	<.001	22.99 (1.50-353.19)	.02
Tested for gonorrhea and/or chlamydia	31% (10/32)	32% (41/127)	.91	2.10 (.40-11.16)	.38
+Gonorrhea	0% (0/10)	5% (2/41)	.48	NA	NA
+Chlamydia	0% (0/10)	15% (6/41)	.20	NA	NA
+Trichomonas	0% (0/3)	10% (1/10)	.57	NA	NA
+Any STI	0% (0/10)	19% (8/42)	.13	NA	NA

Epididymitis, orchitis, or both with a positive urine culture or an STI

Men with epididymitis, orchitis, or both and positive urine culture were significantly older, more likely to be married, had higher urine WBCs, more urine bacteria, higher urine leukocyte esterase, higher urine nitrite, and less likely to be treated for gonorrhea and chlamydia than those with epididymitis, orchitis, or both and positive for an STI (P≤.03 for all) (Table 5). 

**Table 5:** Men with epididymitis and/or orchitis and positive urine culture and no STIs compared to men with epididymitis and/or orchitis and a negative urine culture but infected with gonorrhea, chlamydia, and/or trichomonas.

	+Epididymitis and/or orchitis and +urine culture and -STI (N=32)	+Epididymitis and/or orchitis and -urine culture and +STI (N=20)	p-value
Age	65 (47,77) N= 32	26 (21,30) N= 20	< .001>
Black race, %	63% (20/32)	75% (15/20)	.35
Marital status (% married)	50% (16/32)	0% (0/20)	< .001>
Urine WBCs	101 (41,101)	37 (13,101)	.03
Urine RBCs	11 (3,24)	4 (3,19)	.23
Urine bacteria	1 (1,3)	0 (0,1)	.001
Urine leukocyte esterase	3 (2,3)	1 (0,2)	< .001>
Urine nitrite	34% (11/32)	0% (0/18)	.005
Treated for gonorrhea and chlamydia	28% (9/32)	80% (16/20)	< .001>

Epididymitis, orchitis, or both who were and were not tested for gonorrhea and chlamydia

There were 130 men with epididymitis, orchitis, or both tested for gonorrhea and chlamydia. Among those with epididymitis, orchitis, or both, there were 3.1% (4/129) infected with gonorrhea, 12.3% (16/130) infected with chlamydia, and 6.3% (2/32) infected with trichomonas. Those tested for gonorrhea and chlamydia were significantly younger, more likely Black, unmarried, had fewer urine bacteria and had less urine leukocyte esterase (P≤.04 for all) (Table 6). 

**Table 6:** Comparison of those with epididymitis and/or orchitis who were and were not tested for gonorrhea and chlamydia.

	+Epididymitis and/or orchitis and tested for gonorrhea and chlamydia (N=130)	+Epididymitis and/or orchitis and not tested for gonorrhea and chlamydia (N=126)	p-value
Age	29 (23,40) N= 130	45 (30,63) N= 126	<.001
Black race, %	71% (92/129)	43% (53/124)	<.001
Marital status (% married)	15% (19/105)	35% (44/66)	<.001
Urine WBCs	13 (3,76) N= 69	36 (3,101) N= 82	.24
Urine RBCs	3 (1,13) N= 69	3 (3, 15) N= 82	.10
Urine bacteria	0 (0,1) N= 68	1 (0,2) N= 81	.04
Urine leukocyte esterase	0 (0,1) N= 119	1 (0,3) N= 114	.009
Urine nitrite	4% (5/119)	10% (11/115)	.10
Urine culture growing ≥10,000 CFU/mL bacteriuria	20% (10/51)	20% (22/108)	.91
+Gonorrhea	3% (4/129)	NA	NA
+Chlamydia	12% (16/130)	NA	NA
+Trichomonas	6% (2/32)	50% (1/2)	NA

Men in the dataset who tested positive for an STI and who either had or did not have epididymitis, orchitis, or both

The only significant difference between men in the dataset that tested positive for an STI and did or did not have concurrent epididymitis, orchitis, or both was that the latter were less likely to be of the Black race (P=004) (Table 7).

**Table 7:** Men infected with gonorrhea, chlamydia, and/or trichomonas who either had or did not have epididymitis and/or orchitis.

	+Epididymitis and/or orchitis and positive for gonorrhea, chlamydia, and/or trichomonas (N=20)	+Gonorrhea, chlamydia, and/or trichomonas but no epididymitis and/or orchitis (N=804)	p-value
Age	26 (21,30)	24 (21,30)	.64
Black race, %	75% (15/20)	93% (742/801)	.004
Marital status (% married)	0% (0/20)	4% (36/301)	.56
Urine WBCs	37 (13,101) N= 17	39 (13,101) N= 351	.91
Urine RBCs	4 (3,19) N= 17	3 (2,8) N= 348	.23
Urine bacteria	0 (0,1) N= 17	0 (0,1) N= 350	.64
Urine leukocyte esterase	1 (0,2) N= 18	1 (0,3) N= 420	.67
Diagnosed with a UTI	10% (2/20)	8% (63/804)	.72
Treated for gonorrhea and chlamydia	80% (16/20)	83% (667/804)	.73

Epididymitis, orchitis, or both with and without an STI

Overall, 24.3% (N=761/3132) of men in the dataset tested positive for gonorrhea or chlamydia, and this compares with a rate of 13.8% (18/130) for those diagnosed with epididymitis, orchitis, or both. There were 15.4% (20/130) men with epididymitis, orchitis, or both that tested positive for gonorrhea, chlamydia, or trichomonas. Among those with epididymitis, orchitis, or both and positive for an STI, the rates of infection were 21% (4/20) for gonorrhea, 84% (16/20) for chlamydia, and 60% (3/5) for trichomonas. On univariable analysis, men with an STI were younger and had higher urine WBCs and more leukocyte esterase (P≤.02 for all) (Table 8).

**Table 8:** Men with epididymitis and/or orchitis and infected with a STI compared to men with epididymitis and/or orchitis and testing negative for a STI.

	+Epididymitis and/or orchitis and positive for gonorrhea, chlamydia, and/or trichomonas (N=20)	+Epididymitis and/or orchitis and negative for gonorrhea, chlamydia, and trichomonas (N=110)	p-value
Age	26 (21,30)	31 (23,44)	.02
Black race, %	75% (15/20)	71% (77/109)	.69
Marital status (% married)	0% (0/20)	17% (19/110)	.06
Urine WBCs	37 (13,101) N= 17	13 (3,76) N= 53	.02
Urine RBCs	4 (3,19) N= 17	3 (1,12) N= 53	.19
Urine leukocyte esterase	1 (0, 2) N= 18	0 (0,1) N= 102	.003
Urine nitrite	0% (0/18)	5% (5/102)	.34
Urine culture growing ≥10,000 CFU/mL bacteriuria	0% (0/8)	23% (10/44)	.13
Treated for gonorrhea and chlamydia	80% (16/20)	63% (69/110)	.14
+Gonorrhea	21% (4/19)	0% (0/110)	NA
+Chlamydia	84% (16/19)	0% (0/110)	NA
+Trichomonas	60% (3/5)	0% (0/28)	NA

Epididymitis, orchitis, or both in those treated or not treated for gonorrhea and chlamydia

There were 44.5% (114/256) of men diagnosed with epididymitis, orchitis, or both treated for gonorrhea and chlamydia in the ED. Among those empirically treated, 0% (0/44) had gonorrhea, and 15% (13/85) had chlamydia, which compared to those not empirically treated, of which 5% (4/85) were positive for gonorrhea and 7% (3/45) for chlamydia. On univariable analysis, men who received treatment for epididymitis, orchitis, or both were significantly younger, more likely Black race, and less likely to be married (P<.001 for all) (Table 9). 

**Table 9:** Men with epididymitis and/or orchitis that were treated or not treated for gonorrhea and chlamydia.

	+Epididymitis and/or orchitis and treated for gonorrhea and chlamydia (N=114)	+Epididymitis and/or orchitis and not treated for gonorrhea and chlamydia (N=142)	p-value
Age	29 (23,39)	45 (29,61)	<.001
Black race, %	77% (88/114)	41% (57/139)	<.001
Marital status (% married)	14% (16/113)	33% (47/142)	<.001
Urine WBCs	13 (3,76) N= 67	28 (3,101) N= 84	.79
Urine bacteria	0 (0,1) N= 67	1 (0,1) N= 82	.13
Urine leukocyte esterase	0 (0,1) N= 102	0 (0,2) N= 131	.68
Urine nitrite	8% (8/103)	6% (8/131)	.62
Urine culture growing ≥10,000 CFU/mL bacteriuria	83% (43/52)	79% (84/107)	.54
+Gonorrhea	0% (0/44)	5% (4/85)	.14
+Chlamydia	15% (13/85)	7% (3/45)	.15
+Trichomonas	7% (2/28)	17% (1/6)	.46
+Any STI	19% (16/85)	9% (4/45)	.14

Epididymitis, orchitis, or both and marital status

There were 25% (63/256) married men with epididymitis, orchitis, or both, and 19 were tested for STIs, of which all results were negative. There were six married men with epididymitis, orchitis, or both diagnosed with a UTI, all had urine cultures performed, and four grew bacteria ≥10,000 CFU/mL.

Epididymitis, orchitis, or both in those <35 and ≥35 years of age

Among men with epididymitis, orchitis, or both, 48.4% (124/256) were <35 years of age. Among those <35 years of age, 66.9% (83/124) were tested for gonorrhea and/or chlamydia, of which (19.3% 16/83) were positive for at least one infection, including two with trichomonas, three with gonorrhea, and 14 with chlamydia. 36.6% (15/41) of patients <35 years of age that were not tested for gonorrhea and chlamydia were empirically treated for the infection. Among men <35 years of age, there were 47.6% (59/124) that had a urine culture performed, but only 5.1% (3/59) grew bacteria ≥10,000 CFU/mL. Five men <35 years of age were concurrently diagnosed with a UTI and epididymitis, orchitis, or both, and 0% (0/2) had a positive urine culture ≥10,000 CFU/mL.

In the dataset, 52% (132/256) of men ≥35 years of age were diagnosed with epididymitis, orchitis, or both. There were 22% (7/32) concurrently diagnosed with a UTI, and of these, 67% (4/6) had a positive urine culture ≥10,000 CFU/mL. There were 47 men ≥35 years of age tested for gonorrhea and chlamydia, with 4% (2/47) positive for infection, one patient having both gonorrhea and chlamydia, one having only chlamydia, and only one who had trichomonas. There were 16% (14/85) not tested for gonorrhea or chlamydia and ≥35 years of age that were empirically treated for gonorrhea and chlamydia. 

## Discussion

Epididymitis, orchitis, or both were infrequently diagnosed in men undergoing genitourinary tract laboratory testing in the ED. Similar to previously reported, we found an association between STIs and the patient's age [5,11-14]. For men <35 years of age, STIs are more commonly causing epididymitis, orchitis, or both, with chlamydia being the common pathogen. However, an STI was identified in only 15% of patients with epididymitis, orchitis, or both who underwent testing. STIs were present in men ≥35 years of age; however, they were less frequent, and testing occurred less often in this age group. Testing younger men with epididymitis, orchitis, or both may be because STIs' overall prevalence is higher in younger adults or to age-related bias by the providers. 

*E.coli* was the most frequent bacteria found in urine culture among men diagnosed with epididymitis, orchitis, or both and was more common in men ≥35 years of age. However, only 20.1% of urine cultures were positive. Men ≥35 years of age with epididymitis, orchitis, or both were more likely to be concurrently diagnosed with a UTI and less likely to be tested for an STI-potentially be due to men in this age group being less likely to engage in high-risk sexual behavior and age-related changes such as outlet obstruction from benign prostatic hypertrophy [3,5].

Prior studies have found that the sexual history is frequently not well documented in patients with epididymitis, despite treatment guidelines that suggest patients continue to undergo evaluation for sexually transmitted diseases [26-29]. In our study, 66.9% of patients with epididymitis, orchitis, or both under 35 years of age were tested for STI, 12% were treated for STIs but never tested, and 20% were not tested or treated for STI. Treatment guidelines published by the CDC recommend empiric coverage for sexually transmitted disease in all men with epididymitis ≤35 years of age [4,10]. Sexually transmitted infections also occur in older populations, and high-risk sexual behavior could influence the decision to treat empirically for STI. 

Limits

Data was collected from a single health system in Northeast Ohio, and results may not be generalizable. There was no history and physical exam information available in the dataset. The dataset did not differentiate between acute and chronic epididymitis and orchitis. Patients receiving ceftriaxone or cefixime plus azithromycin or an outpatient prescription for doxycycline could have been treated for infections other than gonorrhea and chlamydia. Because the epididymitis and orchitis diagnoses were combined within the dataset before we examined the data, those patients with only epididymitis or orchitis were not differentiated from those who had epididymitis-orchitis. There was significant variability in infectious screening offered to patients. The study was retrospective, and not all patients with epididymitis, orchitis, or both underwent testing for STIs and received a urine culture. Few men underwent testing for T vaginalis. The STI sampling method (e.g., urethral swab vs. urine sample) may have influenced the sensitivity of diagnosing an STI by NAAT.

## Conclusions

Within our dataset of men in the ED undergoing genitourinary tract laboratory testing, the prevalence of epididymitis, orchitis, or both was only 1.3%. For men <35 years of age, and STI was found in 15.4%, with chlamydia being the most frequently identified STI. There were few men ≥35 years of age tested for an STI, and most of those tested were negative. Only 20.1% of urine cultures from men with epididymitis, orchitis, or both grew bacteria at ≥10,000 CFU/mL. E. coli was the most common bacteria isolated in urine culture. Age ≥35 years and being married were associated with being diagnosed with a UTI and epididymitis, orchitis, or both. Age <35 years, Black race, and being unmarried were associated with an increased likelihood of being tested and treated for an STI.
